# Outdoor air pollution and risk of incident adult haematologic cancer subtypes in a large US prospective cohort

**DOI:** 10.1038/s41416-024-02718-3

**Published:** 2024-05-27

**Authors:** W. Ryan Diver, Lauren R. Teras, Emily L. Deubler, Michelle C. Turner

**Affiliations:** 1 Barcelona Institute for Global Health (ISGlobal), Barcelona, Spain; 2 Universitat Pompeu Fabra (UPF), Barcelona, Spain; 3 Department of Population Science, American Cancer Society, Atlanta, GA USA; 4 CIBER Epidemiología y Salud Pública (CIBERESP), Madrid, Spain

**Keywords:** Risk factors, Cancer epidemiology, Haematological cancer, Epidemiology

## Abstract

**Background:**

Outdoor air pollution and particulate matter (PM) are classified as Group 1 human carcinogens for lung cancer. Pollutant associations with haematologic cancers are suggestive, but these cancers are aetiologically heterogeneous and sub-type examinations are lacking.

**Methods:**

The American Cancer Society Cancer Prevention Study-II Nutrition Cohort was used to examine associations of outdoor air pollutants with adult haematologic cancers. Census block group level annual predictions of particulate matter (PM_2.5_, PM_10_, PM_10-2.5_), nitrogen dioxide (NO_2_), ozone (O_3_), sulfur dioxide (SO_2_), and carbon monoxide (CO) were assigned with residential addresses. Hazard ratios (HR) and 95% confidence intervals (CI) between time-varying pollutants and haematologic subtypes were estimated.

**Results:**

Among 108,002 participants, 2659 incident haematologic cancers were identified from 1992–2017. Higher PM_10-2.5_ concentrations were associated with mantle cell lymphoma (HR per 4.1 μg/m^3^ = 1.43, 95% CI 1.08–1.90). NO_2_ was associated with Hodgkin lymphoma (HR per 7.2 ppb = 1.39; 95% CI 1.01–1.92) and marginal zone lymphoma (HR per 7.2 ppb = 1.30; 95% CI 1.01–1.67). CO was associated with marginal zone (HR per 0.21 ppm = 1.30; 95% CI 1.04–1.62) and T-cell (HR per 0.21 ppm = 1.27; 95% CI 1.00–1.61) lymphomas.

**Conclusions:**

The role of air pollutants on haematologic cancers may have been underestimated previously because of sub-type heterogeneity.

## Background

Ambient air pollution is an established risk factor for multiple diseases and has been recognised as a Group 1 human carcinogen by the International Agency for Research on Cancer (IARC) since 2013 [1]. The primary evidence for carcinogenicity was from studies of lung cancer. Evidence for an association with haematological malignancies was insufficient with mixed results in studies of leukaemias and lymphomas combined and there were a limited number of informative studies for the evaluation.

Studies of ambient air pollution in adults have not consistently identified positive relationships with haematologic cancers. A recent prospective study based on US National Health Interview Survey data reported significant positive associations of average residential census tract PM_2.5_ (fine particulate matter; <2.5 μm in diameter) and Hodgkin lymphoma, non-Hodgkin lymphoma (NHL), and leukaemia mortality [2]. However, findings from other large-scale prospective cohort studies of mortality in the US [3] and Denmark [4] have not reported evidence for associations with PM_2.5_ or other ambient air pollutants. However, a recent pooled study of low-level air pollution in Europe showed associations of NO_2_ with leukaemia and PM_2.5_ with lymphoma [5]. Findings from case-control studies [6–8] have been equally inconsistent though some positive associations were reported among sub-populations.

A major limitation of prior studies is the lack of research on detailed subtypes of haematologic cancers. It is well established that they are a heterogeneous group of diseases which often have distinct risk factors [9]. For example, research on cigarette smoking [9–12] and secondhand smoke [13, 14] has identified associations with Hodgkin lymphoma, peripheral T-cell lymphoma, follicular lymphoma, marginal zone lymphoma, and acute myeloid leukaemia, while observing no associations or even inverse associations with other haematologic cancer subtypes. It is plausible that ambient air pollution may also be associated with some subtypes of haematologic cancer, but not others. This may, in part, explain why previous studies have not consistently observed associations between ambient air pollution and adult haematologic cancers.

In addition, the largest studies to date have been studies of haematologic cancer mortality [2, 3] which makes it unclear whether observed associations are related to the diagnosis of haematologic cancers or rather the likelihood of survival. It is unclear whether associations with less fatal haematologic cancers have been missed. This is particularly important for haematologic cancers since the survival rates vary strongly by sub-type [15].

In this study, we will uniquely examine associations of residential ambient air pollutants (PM_2.5_, PM_10_, PM_10-2.5_, ozone(O_3_), nitrogen dioxide (NO_2_), sulfur dioxide(SO_2_), and carbon monoxide(CO)) with histologic subtypes of haematologic cancers using the Cancer Prevention Study-II Nutrition Cohort, a large prospective study of US men and women with linked data on air pollutants and 25 years of follow-up time. We will extend upon previous mortality-based studies in the overall Cancer Prevention Study-II (CPS-II) here for the first time and examine time-varying average ambient air pollution exposures and cancer incidence endpoints [3].

## Methods

### Study population

Subjects in this analysis were selected from the CPS-II Nutrition Cohort, a prospective study of cancer incidence and mortality in 184,184 men and women from the United States, described in detail elsewhere [16]. Briefly, the Nutrition Cohort is a sub-cohort of the approximately 1.2 million subjects in CPS-II, a prospective study of mortality established by the American Cancer Society in 1982. Participants in the larger study were recruited nationally and completed a four-page questionnaire at enrolment that included residential addresses. CPS-II participants from 21 states with population-based state cancer registries were invited to participate in the Nutrition Cohort in 1992. The goals of this sub-cohort were to obtain updated information on dietary and other exposures and to identify incident cases of cancers. Participants completed a 10-page mailed questionnaire that included information on demographic, medical, behavioural, environmental, occupational, and dietary factors. Follow-up questionnaires were sent to cohort members every 2 years beginning in 1997 through 2017 to ascertain cancer diagnoses and update residential addresses. Responses to follow-up surveys were received from at least 87% of living participants after multiple mailings. All aspects of the CPS-II Nutrition Cohort study have been approved by the Emory University Institutional Review Board. Written informed consent is received from participants to obtain medical records. At the time of each mailed survey, participants are informed that their identifying information is used to link with cancer registries and death indexes.

This analysis excluded subjects from the CPS-II Nutrition Cohort who were lost to follow-up (*n* = 6190), reported a personal history of cancer other than non-melanoma skin cancer at baseline in 1992 (*n* = 22,870), had poor quality address linkage (*n* = 41,225), whose address included a PO Box or “Care of” (*n* = 5383), or reported a diagnosis of cancer in the first survey interval that could not be verified (*n* = 513). The final analytic cohort included 108,002 men and women.

### Outcomes

This analysis includes 2659 subjects with haematologic cancers diagnosed between the date of enrolment (1992/1993) and June 30, 2017. Most cases (*n* = 1907) were identified by self-report of cancer on the follow-up surveys and subsequently verified by medical record abstraction or linkage with state cancer registries. An additional 752 cases were identified as haematologic cancers through automated linkage of the entire cohort with the National Death Index, 78% of these were subsequently verified by linkage with the state cancer registries.

Lymphoid neoplasm subtypes were defined using the Interlymph Pathology Working Group guidelines [17], based on the 2008-revised WHO classification of tumours of haematologic and lymphoid tissues [18]. The International Classification of Disease for Oncology, Second and Third Edition (ICD-O-2 and ICD-O-3) was used to define subtypes with at least 50 cases and included: diffuse large B-cell lymphoma (DLBCL), chronic lymphocytic leukaemia/small lymphocyte lymphoma (CLL/SLL), follicular lymphoma, multiple myeloma, marginal zone lymphoma, mantle cell lymphoma, and T-cell lymphoma. Myeloid leukaemias were divided into acute myeloid leukaemia (AML), and chronic myeloid leukaemia (CML) subtypes.

### Exposure

Ambient air pollution data was obtained from the Centre for Air, Climate and Energy Solutions (CACES) for ambient particulate (PM_2.5_, PM_10_) and gaseous (O_3_, CO, SO_2_, NO_2_) air pollutants at high spatial resolution (estimates at Census block group centroids). Briefly, the CACES modelling approach employed a 3-stage process for each pollutant and year: (1) forward stepwise selection of a subset of ~300 geographic covariates (e.g. land use, roads); (2) partial least squares (PLS) dimension reduction of the selected covariates to obtain ~2–3 composite variables; and 3) universal Kriging employing the composite variables obtained from the PLS. The use of PLS leverages predictive information from a large number of geographic covariates with less concern for model overfitting while also limiting the impact of geographic covariate outliers. Importantly, each pollutant model was developed using the same unified framework. The CACES database includes estimates for O_3_, SO_2_, NO_2_ for the years 1979–2015, PM_10_ for the years 1988–2015, CO for the years 1990–2015, and PM_2.5_ for the years 1999–2015 [19].

Pollutants were linked to the US Census block group of the participants residential addresses. In CPS-II, address data was first collected in 1982, and then updated continuously from 1997–2015. Therefore, address data from 1982 was used for the years 1992–1996 until updating began in 1997. Participants were assigned an average pollutant level for each year based on their address during that year. In a calendar year when participants changed address, the value given for that year was a weighted average based on the number of months at each address. PM_2.5_ values from 1991–1998 were estimated based on the average ratio of PM_10_-PM_2.5_ for each census block group from 1999–2015, as has been done previously [20]. After the estimation of earlier PM_2.5_, data was available for all six pollutants beginning in 1991 (the year prior to the start of follow-up) through 2015. The coarse fraction of PM_10_ was calculated by subtracting PM_2.5_ from PM_10_. Data for 2015 was used for the year 2016 for all pollutants.

### Statistical analysis

In this analysis, ambient air pollutant concentrations at the residences were modelled using yearly time-varying average exposures. Person-years of follow-up for each participant were calculated from the completion of the CPS-II Nutrition Cohort questionnaire in 1992/1993 to date of (1) diagnosis of haematologic cancer; (2) diagnosis of cancer other than haematologic cancer; (3) death occurring between the last returned survey and next mailed survey; 4) return of last questionnaire; 5) last questionnaire the participant was known to be cancer free if they reported haematologic cancer that could not be verified; or 6) end of follow-up on June 30, 2017.

Cox proportional hazards regression [21] was used to compute multivariable-adjusted hazard ratios (HR) and 95% confidence intervals (CI) for the association between each ambient air pollutant and haematologic cancer sub-type incidence. The time scale for the models was assessed in days of follow-up. At each event time of an incident haematologic cancer sub-type diagnosis, a risk set was formed, consisting of all included participants who were not censored, and an average air pollutant exposure was constructed for each member of the risk set from 1991, the year prior to the start of follow-up, to the calendar year prior to the event year based on their residence over follow-up time. Therefore, all exposure data is estimated in the pre-diagnosis period in the statistical models. The HRs were estimated for units representing the distance from the 5^th^ percentile to the mean of each pollutant that subjects were exposed to during follow-up. The proportional hazards assumption was assessed visually and statistically using the cumulative sum of the martingale residuals [22] to identify potential changes in associations over time. Descriptive statistics were calculated showing the distributions of the pollutants, correlations between pollutants, and presenting the mean values by covariate categories.

All models were stratified on single-year of age, and additionally adjusted for sex (male, female), race (white, black, other), education (high school or less, some college, college graduate), marital status (single, married, other), continuous body mass index (BMI), BMI squared, smoking status (never, quit 30+ years, quit 20 to <30 years, quit 10 to <20 years, quit <10 years, current smoker), continuous cigarettes/day and year smoked with squared terms in current smokers, started smoking before age 18 (no, yes), secondhand smoke exposure (h/week), ACS diet score (low, medium, high) [23], alcohol use (non-drinker, <1, 1–2, 2+, and missing drinks/day), an occupational dirtiness index to identify workplace PM_2.5_ exposure [24], and any regular exposure (no/yes/missing) to one of six industrial exposures (asbestos, chemicals/acids/solvents, coal/stone dust, coal tar/pitch/asphalt, formaldehyde, or diesel engine exhaust). Smoking variables were updated time-dependently. Census tract level ecologic covariates from US Census in 1990 and 2000 and the American Community Survey in 2010 were included for median household income, percent college-educated, percent of the population that is African American or Other Race, unemployment rate, and poverty rate and were updated throughout follow-up to account for updated census information over time and residency changes. All *P*-values are two-sided.

Effect modification by sex, smoking status, and region was assessed using a likelihood ratio statistic to compare models with and without multiplicative interaction terms. A *p*-value of <0.05 was used to define statistical significance. Alternative modelling using different covariates (models minimally adjusted for age and sex, models without ecologic variables), exposure assessment based on fixed exposures averaged from 1992–2015, alternate PM_2.5_ exposure data from previous mortality research in CPS-II [3], and two-pollutant models were also conducted. Statistical analysis was conducted using SAS (version 9.4) and R (version 4.2.0). The programme code is available upon request.

## Results

The distribution of air pollutant values at baseline among participants is shown in Table 1. Values in the CPS-II study population are consistent with those observed in the U.S. [19] with average levels below those of the current national ambient air quality standards (NAAQS) [25]. However, there are areas with exposures greater than the current NAAQS. There were high correlations during follow-up for NO_2_ and CO (*r* = 0.74–0.80), and moderate correlations for other pollutants including PM_2.5_ and PM_10_ (*r* = 0.54–0.72), SO_2_ and O_3_ (*r* = 0.50–0.57), NO_2_ and PM_10_ (*r* = 0.41–0.64) (Supplementary Table S1).

**Table 1:** Distribution of average air pollutant values for the CPS-II Nutrition Cohort during follow-up^a^

Variable	Mean	Min	5th	25th	50th	75th	95th	Max	5th-mean difference
PM_2.5_ (µg/m^3^)	12.3	3.3	8.3	10.3	12.1	14.1	17.2	28.8	4.1
PM_10_ (µg/m^3^)	22.1	8.9	15.5	18.8	21.4	24.3	31.7	68.7	6.7
PM_10–2.5_(µg/m^3^)	9.8	1.1	4.8	6.9	9.2	11.7	17.8	41.5	5.0
NO_2_ (ppb)	13.6	1.7	6.5	9.5	12.6	16.5	24.4	51.1	7.2
O_3_ (ppb)	48.2	28.4	38.3	44.6	48.4	52.5	56.7	69.1	9.9
SO_2_ (ppb)	3.8	0.5	1.5	2.3	3.4	4.8	7.4	19.5	2.3
CO (ppm)	0.51	0.07	0.30	0.39	0.47	0.59	0.89	2.00	0.21

^a^Values for pollutants are an average for each participant from baseline to time of event or censoring. The follow-up period of the cohort was from the years 1992–2017 with air pollutant concentrations modelled with a 1-year lag from 1991–2016.

Participants included in this analysis were mostly white (97%) with an average age of 63 years in 1992 and were roughly equally divided among men and women (Table 2). Never-smokers accounted for 43% of the population, although only 9% of participants were currently smoking at baseline. The largest number of participants (62%) lived in the Midwest and Northeastern parts of the United States, however, there was substantial representation from the South (18.3%) and West (19.7%) as well. A majority of subjects did not move from their original census block group over follow-up time (63%) and only 4% had more than three different census block groups during the 25 years of the study.

**Table 2:** Descriptive characteristics and mean pollutant levels of CPS-II Nutrition Cohort participants at baseline^a^

Variable	Categories	Subjects	Pollutant exposure						
		*n* = 108,002	PM_2.5_ (μg/m^3^)	PM_10_ (μg/m^3^)	PM_10-2.5_ (μg/m^3^)	NO_2_ (ppb)	O_3_ (ppb)	SO_2_ (ppb)	CO (ppm)
		*n* (%)	Mean (SD)	Mean (SD)	Mean (SD)	Mean (SD)	Mean (SD)	Mean (SD)	Mean (SD)
Age									
	<55	9203 (8.5)	15.54 (3.69)	27.61 (7.07)	12.07 (5.18)	16.37 (7.46)	52.39 (8.24)	6.15 (2.93)	0.66 (0.30)
	55 to <60	24,077 (22.3)	15.23 (3.77)	27.36 (7.06)	12.13 (5.13)	15.75 (7.24)	51.3 (8.13)	5.85 (2.93)	0.65 (0.29)
	60 to <65	30,220 (28)	15.33 (3.81)	27.52 (7.19)	12.19 (5.19)	15.86 (7.32)	51.16 (8.14)	5.82 (2.97)	0.66 (0.29)
	65 to <70	27,182 (25.2)	15.51 (3.86)	27.84 (7.43)	12.33 (5.38)	15.96 (7.47)	51.06 (8.15)	5.84 (3.01)	0.67 (0.30)
	70+	17,320 (16)	15.59 (3.86)	28.18 (7.76)	12.59 (5.65)	15.92 (7.68)	50.66 (8.29)	5.65 (2.96)	0.68 (0.31)
Sex									
	Male	51,066 (47.3)	15.40 (3.81)	27.64 (7.31)	12.24 (5.31)	15.91 (7.39)	51.26 (8.17)	5.84 (2.96)	0.66 (0.30)
	Female	56,936 (52.7)	15.42 (3.82)	27.71 (7.31)	12.28 (5.30)	15.91 (7.43)	51.13 (8.19)	5.83 (2.98)	0.66 (0.30)
Race									
	White	104,856 (97.1)	15.37 (3.79)	27.56 (7.18)	12.19 (5.22)	15.80 (7.32)	51.23 (8.19)	5.85 (2.96)	0.66 (0.29)
	Black	1581 (1.5)	16.76 (3.38)	29.22 (6.63)	12.46 (4.80)	18.05 (7.68)	51.21 (7.99)	6.36 (3.12)	0.73 (0.31)
	Other	1565 (1.4)	17.03 (5.09)	34.09 (12.00)	17.06 (8.24)	21.00 (10.57)	49.04 (7.96)	4.07 (2.78)	0.93 (0.40)
Education									
	High school or less	32,780 (30.4)	15.20 (3.71)	27.31 (6.71)	12.12 (4.84)	15.02 (7.26)	51.48 (8.10)	6.19 (2.99)	0.62 (0.29)
	Some college	31,265 (28.9)	15.38 (3.93)	28.15 (7.90)	12.77 (5.66)	16.07 (7.68)	50.60 (8.12)	5.56 (2.94)	0.68 (0.31)
	College grad	43,957 (40.7)	15.60 (3.80)	27.62 (7.29)	12.02 (5.34)	16.47 (7.27)	51.40 (8.27)	5.76 (2.94)	0.68 (0.29)
Marital status									
	Single	1656 (1.5)	15.89 (3.42)	28.11 (6.40)	12.23 (4.65)	18.07 (7.92)	50.66 (8.36)	6.35 (3.24)	0.74 (0.32)
	Married	95,480 (88.4)	15.37 (3.82)	27.61 (7.30)	12.24 (5.30)	15.81 (7.34)	51.24 (8.17)	5.83 (2.95)	0.66 (0.30)
	Other	10,866 (10.1)	15.66 (3.78)	28.20 (7.50)	12.53 (5.44)	16.51 (7.87)	50.83 (8.23)	5.79 (3.05)	0.69 (0.31)
Smoking Status									
	Never	46,497 (43.1)	15.40 (3.82)	27.95 (7.48)	12.55 (5.52)	15.71 (7.36)	51.26 (8.13)	5.81 (2.95)	0.66 (0.30)
	Former	50,684 (46.9)	15.43 (3.82)	27.47 (7.23)	12.04 (5.16)	16.09 (7.44)	51.13 (8.23)	5.82 (2.96)	0.66 (0.30)
	Current	9439 (8.7)	15.40 (3.75)	27.45 (6.88)	12.06 (4.88)	15.92 (7.48)	51.24 (8.20)	6.03 (3.06)	0.65 (0.29)
	Other/missing	1382 (1.3)	15.49 (3.63)	27.76 (7.03)	12.26 (5.27)	16.00 (7.43)	51.10 (8.07)	5.98 (3.06)	0.67 (0.30)
Passive smoke exposure									
	No exposure	58,249 (53.9)	15.48 (3.84)	27.93 (7.57)	12.45 (5.54)	16.16 (7.46)	51.16 (8.26)	5.70 (2.95)	0.68 (0.31)
	1 h/week	12,197 (11.3)	15.33 (3.90)	27.50 (7.45)	12.17 (5.35)	15.60 (7.32)	51.00 (8.11)	5.73 (2.89)	0.65 (0.29)
	2–5 h/week	17,116 (15.8)	15.27 (3.76)	27.28 (6.92)	12.01 (4.93)	15.55 (7.33)	51.26 (8.06)	6.00 (3.00)	0.64 (0.29)
	6+ h/week	19,161 (17.7)	15.38 (3.72)	27.37 (6.76)	11.99 (4.80)	15.69 (7.38)	51.38 (8.10)	6.16 (3.03)	0.64 (0.29)
	Missing	1279 (1.2)	15.28 (3.67)	27.70 (6.92)	12.42 (5.14)	15.51 (7.30)	50.85 (8.09)	5.83 (2.91)	0.66 (0.30)
Alcohol use									
	Non-drinker	40,929 (37.9)	15.41 (3.82)	28.12 (7.44)	12.71 (5.53)	15.52 (7.43)	51.36 (8.07)	5.91 (2.96)	0.66 (0.30)
	< 1/day	41,883 (38.8)	15.38 (3.82)	27.36 (7.16)	11.98 (5.07)	16.16 (7.47)	51.16 (8.27)	5.85 (2.98)	0.66 (0.29)
	1–2/day	12,022 (11.1)	15.47 (3.80)	27.36 (7.25)	11.88 (5.16)	16.33 (7.22)	51.09 (8.26)	5.68 (2.95)	0.67 (0.29)
	>2/day	9890 (9.2)	15.48 (3.79)	27.46 (7.41)	11.98 (5.29)	16.15 (7.22)	50.88 (8.23)	5.60 (2.97)	0.67 (0.30)
	Missing	3278 (3.0)	15.40 (3.74)	28.06 (7.25)	12.66 (5.43)	15.34 (7.50)	50.88 (7.98)	5.91 (3.00)	0.66 (0.30)
Body mass index (BMI)									
	<18.5	1373 (1.3)	15.59 (3.94)	28.19 (7.81)	12.61 (5.76)	16.17 (7.28)	50.80 (8.08)	5.55 (2.93)	0.69 (0.30)
	18.5 to <25	47,533 (44.0)	15.48 (3.85)	27.71 (7.37)	12.23 (5.35)	16.12 (7.45)	51.11 (8.21)	5.74 (2.97)	0.67 (0.30)
	25 to <30	42,087 (39.0)	15.37 (3.79)	27.60 (7.27)	12.24 (5.26)	15.77 (7.38)	51.26 (8.19)	5.89 (2.97)	0.65 (0.30)
	30+	15,463 (14.3)	15.32 (3.76)	27.78 (7.27)	12.46 (5.27)	15.68 (7.46)	51.31 (8.09)	5.98 (2.95)	0.65 (0.30)
	Missing	1546 (1.4)	15.29 (3.73)	27.25 (6.49)	11.96 (4.81)	15.43 (6.76)	51.22 (8.16)	6.00 (2.99)	0.64 (0.28)
Diet quality score									
	Low	20,698 (19.2)	15.30 (3.70)	27.36 (6.84)	12.06 (4.93)	15.36 (7.16)	51.36 (8.00)	6.09 (3.03)	0.63 (0.28)
	Medium	63,011 (58.3)	15.37 (3.84)	27.66 (7.38)	12.29 (5.34)	15.94 (7.41)	51.12 (8.21)	5.77 (2.94)	0.66 (0.30)
	High	14,335 (13.3)	15.71 (3.87)	28.09 (7.70)	12.38 (5.64)	16.58 (7.59)	51.36 (8.32)	5.68 (2.94)	0.70 (0.31)
	Missing	9958 (9.2)	15.48 (3.77)	27.86 (7.22)	12.38 (5.28)	15.89 (7.56)	51.06 (8.17)	5.90 (3.02)	0.67 (0.30)
Occupational dirtiness									
	0	70,499 (65.3)	15.46 (3.81)	27.70 (7.23)	12.24 (5.24)	16.07 (7.41)	51.19 (8.21)	5.86 (2.99)	0.67 (0.30)
	1	2493 (2.3)	15.15 (3.84)	27.39 (7.10)	12.24 (5.08)	15.62 (7.28)	50.58 (8.18)	5.72 (3.02)	0.66 (0.30)
	2	22,736 (21.1)	15.44 (3.83)	27.70 (7.54)	12.26 (5.47)	16.01 (7.40)	51.28 (8.15)	5.75 (2.91)	0.67 (0.30)
	3	4101 (3.8)	15.34 (3.75)	27.59 (7.29)	12.25 (5.30)	15.61 (7.40)	51.12 (8.16)	5.92 (3.04)	0.66 (0.30)
	4	4276 (4)	14.77 (3.77)	27.24 (7.29)	12.47 (5.30)	13.82 (7.18)	51.14 (7.93)	5.67 (2.74)	0.59 (0.29)
	5	3219 (3.0)	15.23 (3.84)	27.70 (7.50)	12.47 (5.40)	15.31 (7.45)	51.18 (8.15)	5.93 (3.00)	0.64 (0.30)
	6	678 (0.6)	15.49 (3.84)	28.28 (7.78)	12.79 (5.68)	15.02 (7.27)	51.72 (7.82)	6.55 (3.40)	0.64 (0.30)
Regularly exposed to industrial pollutants									
	Never	73,902 (68.4)	15.45 (3.83)	27.71 (7.33)	12.26 (5.31)	16.07 (7.42)	51.18 (8.21)	5.80 (2.95)	0.67 (0.30)
	Ever	23,496 (21.8)	15.37 (3.82)	27.71 (7.49)	12.34 (5.41)	15.70 (7.53)	51.29 (8.15)	5.86 (2.99)	0.66 (0.31)
	Missing	10,604 (9.8)	15.27 (3.69)	27.39 (6.73)	12.12 (4.97)	15.23 (7.06)	51.06 (8.04)	6.04 (3.03)	0.65 (0.29)
Live in rural area									
	No	98,371 (91.1)	15.64 (3.79)	27.98 (7.45)	12.33 (5.41)	16.66 (7.31)	51.33 (8.27)	5.87 (3.00)	0.68 (0.30)
	Yes	9631 (8.9)	13.05 (3.18)	24.62 (4.77)	11.57 (3.91)	8.24 (2.28)	49.77 (7.12)	5.50 (2.63)	0.43 (0.16)
U.S. Region									
	Northeast	32,788 (30.4)	15.98 (2.66)	25.72 (4.33)	9.74 (2.78)	17.51 (7.23)	57.10 (5.45)	8.7 (2.31)	0.65 (0.27)
	South	19,800 (18.3)	16.34 (3.81)	26.12 (2.93)	9.78 (2.71)	13.72 (4.85)	51.31 (10.32)	5.41 (2.41)	0.55 (0.18)
	Midwest	34,112 (31.6)	13.44 (3.02)	25.39 (3.88)	11.95 (2.44)	12.80 (4.84)	47.35 (5.90)	5.08 (1.94)	0.51 (0.16)
	West	21,302 (19.7)	16.84 (5.01)	35.80 (11.30)	18.96 (7.33)	20.46 (9.70)	48.15 (7.23)	3.03 (1.85)	1.02 (0.30)

^a^Baseline data for participant characteristics and the start of outcome follow-up is in 1992, however, the pollutant exposure data lagged by 1 year to 1991.

Only modest differences in air pollution exposure at baseline were observed by participant characteristics (Table 2). There was some indication of higher PM_10_ values for older participants, while the inverse was seen for O_3_ and SO_2_. Non-white participants generally lived in areas with higher levels of all pollutants. We did not observe large differences in pollutant exposure by lifestyle factors such as smoking, alcohol, and BMI. Subjects living in urban areas had higher pollutant exposures. There were also regional differences in residential air pollution exposures.

There was generally no clear association of ambient air pollution with NHL and myeloid leukaemia overall (Table 3). There were some positive associations between several air pollutants and Hodgkin lymphoma, with a statistically significant association observed for NO_2_ (HR per 7.2 ppb = 1.39, 95% CI 1.01–1.92).

**Table 3:** Association of air pollutants with haematologic subtypes in the CPS-II Nutrition cohort from 1992–2017

Cancer site	Person-years	Cases	Hazard ratios^a^ (95% confidence intervals)						
			PM_2.5_ per 4.1 μg/m^3^	PM_10_ per 6.7 μg/m^3^	PM_10-2.5_ per 5.0 μg/m^3^	NO_2_ per 7.2 ppb	O_3_ per 9.9 ppb	SO_2_ per 2.3 ppb	CO per 0.21 ppm
Hodgkin lymphoma	1,648,416	54	1.30 (0.88–1.92)	1.12 (0.80–1.56)	0.99 (0.70–1.40)	1.39 (1.01–1.92)	1.39 (0.88–2.21)	1.23 (0.95–1.60)	1.25 (0.94–1.65)
Non-Hodgkin lymphomas	1,648,416	2276	0.94 (0.88–1.00)	0.95 (0.89–1.00)	0.97 (0.92–1.03)	0.99 (0.93–1.05)	0.95 (0.88–1.02)	0.94 (0.90–0.99)	1.03 (0.98–1.09)
B-cell lymphomas	1,648,416	2069	0.94 (0.88–1.00)	0.95 (0.89–1.01)	0.97 (0.92–1.03)	0.99 (0.93–1.05)	0.94 (0.87–1.01)	0.93 (0.88–0.98)	1.03 (0.98–1.09)
DLBCL	1,648,416	473	0.94 (0.82–1.08)	0.97 (0.85–1.09)	0.99 (0.88–1.12)	1.03 (0.90–1.17)	1.06 (0.90–1.24)	0.95 (0.85–1.07)	1.07 (0.96–1.20)
CLL/SLL	1,648,416	507	0.88 (0.77–1.01)	0.86 (0.76–0.98)	0.90 (0.80–1.02)	0.84 (0.74–0.96)	0.94 (0.81–1.09)	0.90 (0.81–1.00)	0.91 (0.82–1.02)
Follicular	1,648,416	269	1.04 (0.86–1.25)	0.98 (0.83–1.15)	0.95 (0.80–1.12)	1.02 (0.86–1.21)	1.02 (0.83–1.25)	0.95 (0.82–1.10)	1.10 (0.95–1.27)
Multiple myeloma	1,648,416	434	0.92 (0.79–1.07)	0.87 (0.76–1.00)	0.89 (0.78–1.02)	1.02 (0.89–1.17)	0.91 (0.78–1.07)	0.94 (0.84–1.06)	1.03 (0.91–1.16)
Marginal zone	1,648,416	114	0.98 (0.73–1.31)	1.14 (0.89–1.46)	1.19 (0.94–1.51)	1.30 (1.01–1.67)	0.84 (0.61–1.14)	0.96 (0.76–1.21)	1.30 (1.04–1.62)
Mantle cell	1,648,416	71	0.91 (0.63–1.33)	1.29 (0.95–1.74)	1.43 (1.08–1.90)	1.05 (0.75–1.48)	0.76 (0.51–1.13)	0.79 (0.57–1.08)	1.09 (0.80–1.47)
Other B-cell	1,648,416	201	0.99 (0.80–1.23)	1.04 (0.86–1.26)	1.05 (0.88–1.27)	1.04 (0.85–1.26)	0.77 (0.61–0.97)	0.96 (0.81–1.13)	1.01 (0.84–1.20)
T-cell lymphomas	1,648,416	98	0.84 (0.61–1.16)	0.94 (0.70–1.26)	1.03 (0.78–1.35)	1.08 (0.81–1.44)	0.97 (0.70–1.36)	0.88 (0.68–1.13)	1.27 (1.00–1.61)
Other NHL	1,648,416	109	1.05 (0.79–1.40)	0.99 (0.75–1.29)	0.95 (0.73–1.23)	0.96 (0.73–1.27)	1.15 (0.84–1.58)	1.19 (0.99–1.44)	0.90 (0.70–1.15)
Myeloid leukaemias	1,648,416	329	1.00 (0.84–1.18)	0.99 (0.85–1.15)	0.99 (0.85–1.15)	0.96 (0.82–1.13)	1.09 (0.91–1.32)	1.01 (0.89–1.15)	0.95 (0.83–1.10)
AML	1,648,416	223	0.98 (0.80–1.20)	0.94 (0.78–1.13)	0.93 (0.78–1.12)	0.96 (0.79–1.16)	1.02 (0.82–1.28)	1.07 (0.92–1.24)	0.95 (0.79–1.13)
CML	1,648,416	61	0.92 (0.62–1.37)	1.13 (0.80–1.60)	1.22 (0.88–1.69)	0.77 (0.51–1.14)	1.33 (0.86–2.07)	0.86 (0.62–1.19)	0.85 (0.60–1.22)

^a^Stratified by 1-year age in 1992 and adjusted for sex, race, education, marital status, BMI, smoking status, years smoked, cigarettes/day, years since quit smoking, started smoking before the age of 18, secondhand smoke exposure, diet, alcohol, occupational dirtiness, industrial exposures, and census tract data (median household income, % college educated, % African American, % other non-White race, unemployment rate, and poverty rate).

When examining more detailed NHL subtypes (Table 3) there were some positive associations of residential particulate matter with marginal zone and mantle cell lymphoma. There was a statistically significant association between PM_10-2.5_ and mantle cell lymphoma (HR per 5 μg/m^3^ = 1.43; 95% CI 1.08–1.90). Among the gaseous pollutants, there were some positive associations observed with NO_2_ and CO; associations with marginal zone lymphoma (NO_2_ HR per 7.2 ppb = 1.30, 95% CI 1.01–1.67; CO HR per 0.21 ppm = 1.30, 95% CI 1.04–1.62) and T-cell lymphoma (CO HR per 0.21 ppm = 1.27, 95% CI 1.00–1.61) were statistically significant. Other NHL subtypes were generally not associated with any other pollutants. There were also some inverse associations observed, including of both PM_10_ and NO_2_ with CLL/SLL.

Alternative adjustment with a minimal set of covariates (age and sex) or without the ecologic covariates were similar (Supplemental Table 2), as were models that used fixed (non-time varying) air pollutant estimates for comparison (Supplemental Table 3).

**Fig. 1:**
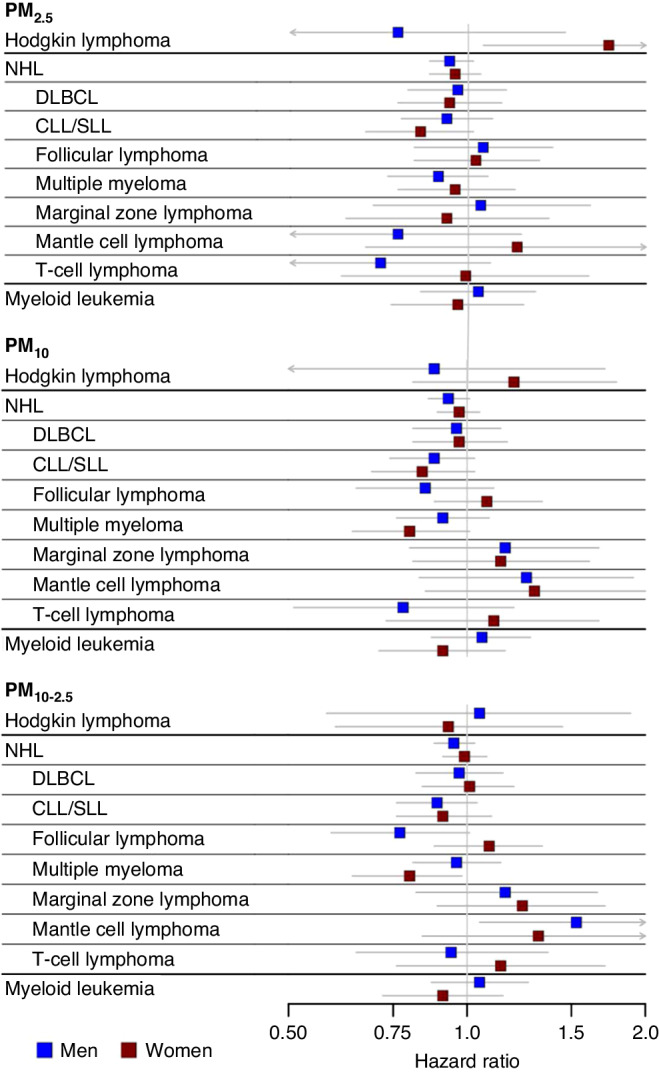
Associations of particulate matter air pollutants and risk of incident haematologic cancer subtypes by sex. Hazard ratios for PM_2.5_, PM_10_, and PM_10-__2.5_ are shown in three panels with separate indicators for men (blue) and women (red) with 95% confidence intervals shown as gray lines. Confidence limits that extend beyond the scale are indicated by arrowheads. Units for the HRs: PM_2.5_ (4.1 µg/m^3^), PM_10_ (6.7 µg/m^3^), and PM10-2.5 (5 µg/m^3^).

**Fig. 2:**
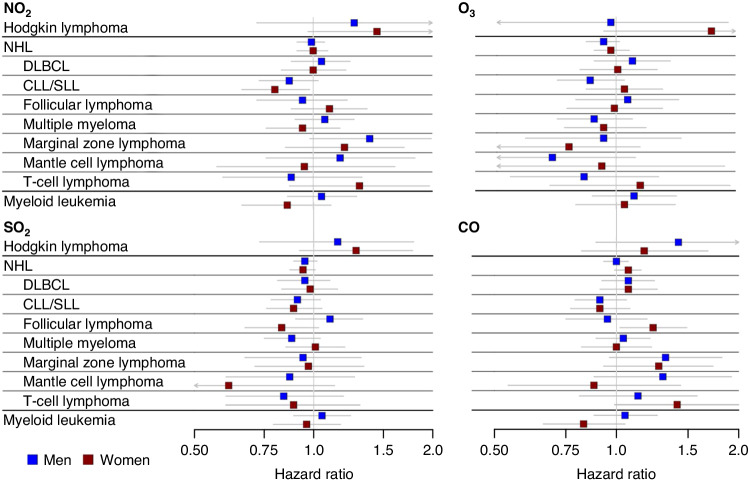
Associations of gaseous air pollutants and risk of incident haematologic cancer subtypes by sex. Hazard ratios for NO_2_, O_3_, SO_2_, and CO are shown in four panels with separate indicators for men (blue) and women (red) wiith 95% confidence intervals shown as gray lines. Confidence limits that extend beyond the scale are indicated by arrowheads. Units for the HRs: NO_2_ (7.2 ppb), O_3_ (9.9 ppb), SO_2_ (2.3 ppb), and CO (0.21 ppm).

Some differences by sex were observed (Figs. 1 and 2; Supplemental Tables 4 and 5). PM_2.5_ was associated with a higher risk of Hodgkin lymphoma (HR per 4.1 μg/m^3^ = 1.73; 95% CI 1.06–2.82) in women. The association was not present in men, and the test for interaction was statistically significant (p-int = 0.02). CO was associated with an increased risk of follicular lymphoma (HR per 0.21 ppm = 1.23; 95%CI 1.02–1.49) in women, but not men.

Results in never-smokers are shown in Table 4. The statistically significant positive association between PM_2.5_ and the risk of Hodgkin lymphoma that was observed in women was also present in never-smokers. The association of NO_2_ and risk of Hodgkin lymphoma was also elevated (HR per 7.2 ppb = 1.58, 95% CI 0.96–2.61), but no longer statistically significant in never-smokers. Associations with air pollutants and other subtypes in never-smokers were generally in the same direction as the models including all subjects, but some results were no longer statistically significant.

**Table 4:** Association of air pollutants with haematologic subtypes in never-smokers in the CPS-II Nutrition Cohort from 1992–2017.

Cancer site	Person-years	Cases	Hazard ratios^a^ (95% confidence intervals)						
			PM_2.5_ per 4.1 μg/m^3^	PM_10_ per 6.7 μg/m^3^	PM_10-2.5_ per 5.0 μg/m^3^	NO_2_ per 7.2 ppb	O_3_ per 9.9 ppb	SO_2_ per 2.3 ppb	CO per 0.21 ppm
Hodgkin lymphoma	768,366	24	1.93 (1.10–3.38)	1.35 (0.83–2.19)	0.96 (0.56–1.65)	1.58 (0.96–2.61)	1.67 (0.83–3.37)	1.23 (0.86–1.77)	1.19 (0.76–1.86)
Non-Hodgkin lymphomas	768,366	979	0.95 (0.86–1.05)	0.97 (0.89–1.06)	0.99 (0.91–1.08)	1.01 (0.92–1.10)	0.98 (0.88–1.09)	0.97 (0.90–1.05)	1.06 (0.98–1.14)
B-cell lymphomas	768,366	903	0.95 (0.86–1.06)	0.95 (0.87–1.04)	0.97 (0.89–1.05)	1.00 (0.91–1.10)	0.98 (0.87–1.09)	0.97 (0.90–1.05)	1.06 (0.98–1.15)
DLBCL	768,366	215	0.91 (0.74–1.12)	0.97 (0.81–1.17)	1.02 (0.86–1.21)	1.02 (0.84–1.23)	1.10 (0.87–1.38)	0.97 (0.83–1.15)	1.12 (0.96–1.32)
CLL/SLL	768,366	197	0.90 (0.72–1.12)	0.87 (0.71–1.06)	0.90 (0.74–1.08)	0.78 (0.62–0.96)	0.95 (0.75–1.21)	0.88 (0.74–1.06)	0.93 (0.77–1.11)
Follicular	768,366	118	1.22 (0.92–1.61)	1.22 (0.96–1.55)	1.14 (0.90–1.44)	1.27 (0.98–1.63)	1.11 (0.81–1.51)	0.97 (0.78–1.21)	1.19 (0.95–1.48)
Multiple myeloma	768,366	208	0.84 (0.67–1.04)	0.81 (0.66–0.99)	0.86 (0.71–1.04)	0.97 (0.79–1.19)	0.89 (0.70–1.12)	0.88 (0.74–1.06)	1.01 (0.85–1.21)
Marginal zone	768,366	49	1.17 (0.76–1.81)	0.96 (0.65–1.43)	0.87 (0.59–1.27)	1.30 (0.90–1.88)	0.99 (0.62–1.58)	1.28 (0.95–1.71)	1.18 (0.85–1.64)
Mantle cell	768,366	26	0.90 (0.49–1.65)	1.16 (0.71–1.88)	1.26 (0.79–2.01)	1.02 (0.60–1.76)	0.99 (0.49–2.01)	1.01 (0.61–1.67)	1.05 (0.63–1.73)
Other B-cell	768,366	90	1.07 (0.77–1.48)	1.06 (0.80–1.40)	1.03 (0.78–1.35)	1.15 (0.87–1.53)	0.82 (0.59–1.16)	1.15 (0.91–1.45)	1.11 (0.86–1.42)
T-cell lymphomas	768,366	41	0.73 (0.43–1.22)	1.10 (0.71–1.70)	1.30 (0.88–1.90)	0.98 (0.62–1.56)	0.99 (0.59–1.67)	0.77 (0.50–1.19)	1.23 (0.84–1.80)
Other NHL	768,366	35	1.19 (0.70–2.01)	1.37 (0.89–2.10)	1.34 (0.88–2.02)	1.09 (0.67–1.78)	0.95 (0.54–1.68)	1.25 (0.87–1.78)	0.90 (0.57–1.43)
Myeloid leukaemias	768,366	122	0.85 (0.64–1.12)	0.86 (0.67–1.11)	0.92 (0.72–1.17)	0.88 (0.67–1.16)	0.99 (0.73–1.34)	1.02 (0.82–1.27)	0.85 (0.66–1.09)
AML	768,366	85	0.83 (0.59–1.17)	0.83 (0.61–1.14)	0.89 (0.67–1.20)	0.81 (0.58–1.13)	0.83 (0.58–1.20)	1.09 (0.84–1.41)	0.81 (0.60–1.09)
CML	768,366	25	0.78 (0.41–1.47)	0.82 (0.45–1.50)	0.92 (0.53–1.60)	1.04 (0.59–1.86)	1.38 (0.69–2.75)	0.88 (0.52–1.48)	0.94 (0.56–1.59)

^a^Stratified by 1-year age in 1992 and adjusted for sex, race, education, marital status, BMI, secondhand smoke exposure, diet, alcohol, occupational dirtiness, industrial exposures, and census tract data (median household income, % college educated, % African American, % other non-White race, unemployment rate, and poverty rate).

There were no strong regional differences in PM_2.5_ associations with haematologic cancers (Supplemental Table 6). Statistically significant associations were largely unchanged in selected two-pollutant models (Supplemental Table 7).

## Discussion

We conducted an analysis of ambient air pollutants and the risk of incident haematologic cancers and found significant positive associations with some subtypes. Higher coarse particulate matter exposure was positively associated with mantle cell lymphoma risk, while fine particulate matter was associated with Hodgkin lymphoma in women and never-smokers. Among gaseous pollutants, increased NO_2_ was associated with Hodgkin lymphoma. We also observed an increased risk of marginal zone lymphoma with higher levels of CO, and similar associations with follicular lymphoma and T-cell lymphoma that were limited to women. Several patterns emerged as the main findings of this study. Ambient air pollutants were generally associated with a higher risk of Hodgkin lymphoma, CO was positively associated with multiple NHL subtypes, and some previously unstudied NHL subtypes were found to be associated with ambient air pollution. This suggests that ambient air pollution may play a larger role in haematologic cancer risk than previously observed.

Most previous research on outdoor air pollutants and haematologic cancers has focused on broad groups that do not account for aetiologic heterogeneity. Common groups examined include “leukaemia” [2–6, 8, 26, 27], “leukaemia and lymphoma” [28], “haematologic cancer” [29], or “non-Hodgkin lymphomas (NHL)” [2, 3, 5]. Of the prospective cohorts, one large US study of PM_2.5_ and fatal cancers found increased risks of leukaemia and NHL [2], as did a pooled European cohort of NO_2_ with leukaemia and PM_2.5_ with lymphoma [5]. However, another large US study found no associations with PM_2.5_, NO_2_, or O_3_ for the same sites [3]. A large cohort study of cancer risk in Denmark also found no association with nitrogen oxides (NOx) and leukaemia or NHL [4], nor did a smaller US cohort study of total suspended particles with leukaemias and lymphomas[28]. Large registry-based case-control studies of leukaemia in Denmark identified significant associations with PM_2.5_ [6, 27], while ecologic studies found associations with some pollutants, but not others [29, 30]. Given the differences we observed by haematologic cancer sub-type, the inconsistent or null findings from these studies may be explained by these broad groupings of all lymphomas or leukaemias together, thus masking associations with one or more subtypes.

The observed associations with particulate matter and Hodgkin lymphoma in this study are consistent with previous research. PM_2.5_ was positively associated with Hodgkin lymphoma with the strongest findings in women and never-smokers, though there were only 54 total observed cases. A population-based case-control study in Denmark [7] found no overall association with Hodgkin lymphoma, but there was an elevated risk of the classical Hodgkin lymphoma sub-type for PM_2.5_ per 5 μg/m^3^ (HR = 1.21, 95%CI 0.96–1.54). Low-level exposure to PM_2.5_ in Europe [5] showed an elevated although non-significant association with Hodgkin lymphoma (HR per 5 μg/m^3^ = 1.31, 95%CI 0.79–2.16). A large US cohort study of fatal cancer [2] identified a statistically significant association of 10 μg/m^3^ of PM_2.5_ with a higher risk of Hodgkin lymphoma (HR = 4.18, 95%CI 1.20–14.60). There was a weakly elevated but non-significant association between PM_2.5_ and fatal Hodgkin lymphoma in the larger CPS-II mortality cohort [3] (HR per 4.4 μg/m^3^ = 1.12 95% CI 0.82–1.54). In sensitivity analyses using PM_2.5_ exposure data from the previous mortality study, there were similar elevated HRs for Hodgkin lymphoma as observed here (Supplemental Table 8). Our findings in women were not examined in other studies, but the stronger association in never-smokers was also seen in the one study that evaluated smoking [2]. The PM_2.5_ and Hodgkin lymphoma association may be more apparent in never-smokers if there was residual confounding due to cigarette smoking. This may also explain the observed stronger associations in women since half are never-smokers compared to only 32% of men, although results may also be due to chance. Overall, there is some emerging evidence for an association between PM_2.5_ and Hodgkin lymphoma.

We observed some associations with particulate matter and haematologic subtypes that are not as well studied in the literature. Our finding of an association between coarse particulate matter and the risk of mantle cell lymphoma has not been previously reported. To our knowledge, studies of potential risk factors for mantle cell lymphoma are relatively few, and suggested risk factors remain unconfirmed [31]. A study in Denmark found that PM_2.5_ was associated with an increased risk of the AML sub-type [6], which we did not observe. The novel association with particulate matter and mantle cell lymphoma requires additional follow-up.

Gaseous pollutants have not been well studied in relation to haematologic subtypes, and we observed some novel findings. In this study, we observed an association between NO_2_ (an indicator of vehicular traffic emissions) and an increased risk of Hodgkin lymphoma. In a previous mortality analysis in CPS-II, there was a positive, but imprecise association of NO_2_ and fatal Hodgkin lymphoma (HR per 6.5 ppb = 1.16, 95% CI 0.87–1.53, *n* = 125 deaths). Among other studies of Danish adults [7] and Swiss children [32] there was no association with NO_2_. A recent meta-analysis suggested an association of a threshold effect at higher levels of NO_2_ exposure with acute lymphocytic leukaemia (ALL) in children [33], but no association with PM_2.5_ or PM_10_. In a US case-control study, children diagnosed with ALL were more likely to have mothers living in areas with higher levels of CO based on traffic [34]. However, ALL is relatively rare in adults, and we were unable to examine it in this population. The statistically significant positive associations for CO with marginal zone lymphoma, T-cell lymphoma, and women with follicular lymphoma are supported by other epidemiologic evidence related to tobacco smoke and have biological plausibility.

Epidemiologic research on tobacco smoke is informative for associations with air pollutants because tobacco smoke is a meaningful source of particulate matter and CO [35]. The large Interlymph consortium evaluated NHL risk factors by sub-type and found more years of cigarette smoking to be associated with increased risk of follicular, marginal zone, mantle cell, and T-cell lymphomas while other subtypes had associations with smoking in inverse directions [9]. Studies on secondhand smoke also showed increased associations with follicular lymphoma [13, 14]. There is also evidence suggesting that the tobacco smoking associations with follicular lymphoma are stronger in women [10], similar to what we observed with CO. Increases in circulating oestrogens related to smoking [36] can lead to poor precursor B-cell differentiation and an accumulation of non-cycling cells that could develop into NHL [37]. If this is being driven by CO, then this pathway may be more relevant for women with existing oestrogen levels that are already higher than men. Studies of tobacco smoke show support for the observed associations with CO exposure with specific NHL subtypes in this study.

There are plausible mechanisms for the observed associations with air pollutants and haematologic cancers. CO directly interacts with the haematologic system including binding with haemoglobin to form carboxyhemoglobin which can induce hypoxia in tissues. CO is also involved in the co-regulation of oxidative stress and reduces apoptosis which could be associated with a higher risk of cancer, however, it also has weak anti-inflammatory and antiproliferative effects [38]. In animal studies, mice exposed to urban air were found to have a statistically significant increase in micronuclei frequency in lymphocytes that was positively correlated with CO and PM_2.5_ [39]. Human studies on ambient CO as a carcinogen have primarily been on lung cancer, however, this study and supporting animal and mechanistic work suggest that future work should examine its role in haematologic cancers. For Hodgkin lymphoma, PM_2.5_ has been associated with inflammation [40] which may play a role in the reactivation of the Epstein–Barr virus and the development of Hodgkin lymphoma [41]. Beyond direct effects, it is also possible that the measured pollutants in this study are markers for established carcinogens such as polyaromatic hydrocarbons (PAHs) or dioxins [42]. The burning of fossil fuels and biomass are sources of pollutants included in the analysis (PM_2.5_, NO_2_, SO_2_, and CO) as well as pollutants like PAHs and dioxins that were not. Therefore, associations with CO may represent a better assessment of exposure to these types of pollutant sources. There was some suggestion of inverse associations with pollutants and CLL/SLL in this and other studies [4, 8], but we did not identify any strong biological reasons for these findings indicating they may be due to chance.

The strengths of this study include its large nation-wide prospective design with the ability to examine multiple subtypes of incident haematologic cancer with time-varying air pollution exposure estimates. We are not aware of other studies of air pollution that have comprehensively examined the subtypes of NHL, and some new potential associations have been observed here which should be examined in further work. The updating of air pollution data over-time allowed us to examine pre-diagnosis pollutants ensuring appropriate temporality, and to account for location changes. Additionally, data was linked to census block groups which are fine areas of geospatial resolution. Detailed individual-level data allowed for control of confounders and the large sample size allowed for stratification by important factors such as sex and smoking status which was also updated over follow-up time.

This study is limited in its racial and ethnic diversity, and we are not able to observe whether air pollution plays a role in known haematologic cancer rate differences by race. The study population is also older, and it is possible we are missing important associations in a younger adult group. Approximately half of all lymphoid malignancies are diagnosed after 65, however, particular subtypes like Hodgkin lymphoma and precursor lymphoid leukaemia/lymphoma are primarily diagnosed in early adulthood or childhood [15]. Despite the study’s large size overall, when examining the rarer subtypes, we remain limited in statistical power and may be missing important associations, or results that were observed may be due to chance. Additionally, many air pollutants are correlated and it is possible observed associations are the effect of a mixture of multiple pollutants, which was not addressed in the current study. Mixtures of air pollutants are an important area of future research.

In conclusion, this study identified several novel associations of air pollutants with incident haematologic cancer subtypes. The sub-type-specific findings may explain mixed associations found with larger groupings of haematologic cancers in previous research. It will be important for future studies to replicate these findings and may require pooled efforts to have an adequate sample size for the rarer subtypes. These findings suggest that the role of air pollutants in the risk of haematologic cancers may have been underestimated previously because subtypes were not accounted for.

### Supplementary information

Supplemental Material

## Data Availability

The data underlying the findings of this study are restricted by the Emory University Institutional Review Board, which approved the consent forms. Data are available from the American Cancer Society by following the ACS Data Access Procedures (https://www.cancer.org/content/dam/cancer-org/research/epidemiology/cancer-prevention-study-data-access-policies.pdf) for researchers who meet the criteria for access to confidential data.
