# Supplementary material for: Outdoor air pollution and risk of incident adult haematologic cancer subtypes in a large US prospective cohort
Source: Br J Cancer. 2024 May 27;131(1):149–58. doi: 10.1038/s41416-024-02718-3 (PMC11231250; doi:10.1038/s41416-024-02718-3)
Supplement: Supplementary file 1 — Supplemental Material [file 41416_2024_2718_MOESM1_ESM.docx]

Supplemental Table 1. Pearson Correlation Coefficients among pollutants for participants remaining in the study at different time-points of follow-up.

|  | ***Baseline^1^*** | | | | | | |
| --- | --- | --- | --- | --- | --- | --- | --- |
|  | PM_2.5_ | PM_10_ | PM_10-2.5_ | NO_2_ | O_3_ | SO_2_ | CO |
| PM_2.5_ | 1.00 |  |  |  |  |  |  |
| PM_10_ | 0.72 | 1.00 |  |  |  |  |  |
| PM_10-2.5_ | 0.27 | 0.86 | 1.00 |  |  |  |  |
| NO_2_ | 0.56 | 0.64 | 0.48 | 1.00 |  |  |  |
| O_3_ | 0.51 | 0.14 | -0.16 | 0.27 | 1.00 |  |  |
| SO_2_ | 0.26 | -0.11 | -0.35 | 0.14 | 0.57 | 1.00 |  |
| CO | 0.40 | 0.64 | 0.59 | 0.79 | -0.01 | -0.09 | 1.00 |
|  | ***Middle of follow-up after address updating begins^1^*** | | | | | | |
| PM_2.5_ | 1.00 |  |  |  |  |  |  |
| PM_10_ | 0.56 | 1.00 |  |  |  |  |  |
| PM_10-2.5_ | -0.08 | 0.78 | 1.00 |  |  |  |  |
| NO_2_ | 0.35 | 0.49 | 0.32 | 1.00 |  |  |  |
| O_3_ | 0.50 | 0.07 | -0.29 | 0.01 | 1.00 |  |  |
| SO_2_ | 0.51 | 0.01 | -0.37 | 0.16 | 0.50 | 1.00 |  |
| CO | 0.11 | 0.38 | 0.38 | 0.80 | -0.18 | -0.06 | 1.00 |
|  | ***Last full year of follow-up^1^*** | | | | | | |
| PM_2.5_ | 1.00 |  |  |  |  |  |  |
| PM_10_ | 0.54 | 1.00 |  |  |  |  |  |
| PM_10-2.5_ | 0.22 | 0.94 | 1.00 |  |  |  |  |
| NO_2_ | 0.53 | 0.41 | 0.26 | 1.00 |  |  |  |
| O_3_ | 0.40 | 0.20 | 0.07 | 0.47 | 1.00 |  |  |
| SO_2_ | 0.12 | 0.03 | -0.01 | -0.08 | -0.08 | 1.00 |  |
| CO | 0.29 | 0.29 | 0.22 | 0.74 | 0.35 | -0.02 | 1.00 |

^1^Follow-up for this study was from 1992-2017. Baseline is 1992, middle of follow-up is 1999, and last full year is 2016. Due to the 1-year lag, pollutant data from 1991,1998, and 2015 are presented.

Supplemental Table 2. Alternative covariate models for the association of air pollutants with hematologic subtypes in the CPS-II Nutrition Cohort from 1992-2017.

|  |  |  |  | **Hazard Ratios (95% Confidence Intervals)** | | | | | | |
| --- | --- | --- | --- | --- | --- | --- | --- | --- | --- | --- |
|  |  |  |  | **PM_2.5_** | **PM_10_** | **PM_10-2.5_** | **NO_2_** | **O_3_** | **SO_2_** | **CO** |
|  | **Cancer Site** | **Person-years** | **Cases** | **per 4.1 μg/m^3^** | **per 6.7 μg/m^3^** | **per 5.0 μg/m^3^** | **per 7.2 ppb** | **per 9.9 ppb** | **per 2.3 ppb** | **per 0.21 ppm** |
| **Minimallly^1^ Adjusted** | Hodgkin Lymphoma | 1,648,416 | 54 | 1.34 (0.93-1.94) | 1.15 (0.86-1.54) | 1.02 (0.75-1.39) | 1.33 (1.02-1.73) | 1.41 (0.91-2.19) | 1.21 (0.94-1.55) | 1.20 (0.95-1.50) |
|  | NHL | 1,648,416 | 2,276 | 0.94 (0.88-1.00) | 0.96 (0.91-1.01) | 0.98 (0.93-1.03) | 0.99 (0.95-1.05) | 0.94 (0.88-1.00) | 0.94 (0.90-0.99) | 1.03 (0.98-1.07) |
|  | B-Cell Lymphomas | 1,648,416 | 2,069 | 0.94 (0.88-1.00) | 0.96 (0.91-1.02) | 0.99 (0.94-1.04) | 1.00 (0.95-1.05) | 0.93 (0.86-1.00) | 0.93 (0.88-0.98) | 1.03 (0.99-1.08) |
|  | DLBCL | 1,648,416 | 473 | 0.94 (0.82-1.07) | 0.99 (0.88-1.11) | 1.02 (0.92-1.14) | 1.04 (0.94-1.16) | 1.02 (0.88-1.19) | 0.93 (0.84-1.04) | 1.08 (0.98-1.19) |
|  | CLL/SLL | 1,648,416 | 507 | 0.89 (0.78-1.01) | 0.89 (0.79-1.00) | 0.93 (0.83-1.04) | 0.91 (0.81-1.01) | 0.93 (0.81-1.08) | 0.90 (0.81-0.99) | 0.96 (0.88-1.06) |
|  | Follicular | 1,648,416 | 269 | 1.00 (0.84-1.19) | 0.98 (0.84-1.14) | 0.97 (0.84-1.13) | 0.97 (0.83-1.12) | 0.99 (0.81-1.21) | 0.94 (0.82-1.08) | 1.04 (0.92-1.17) |
|  | Multiple Myeloma | 1,648,416 | 434 | 0.92 (0.80-1.06) | 0.90 (0.79-1.02) | 0.92 (0.82-1.04) | 1.00 (0.89-1.12) | 0.91 (0.78-1.06) | 0.94 (0.84-1.06) | 1.02 (0.92-1.12) |
|  | Marginal Zone | 1,648,416 | 114 | 0.96 (0.73-1.27) | 1.11 (0.89-1.39) | 1.16 (0.94-1.42) | 1.19 (0.97-1.46) | 0.82 (0.60-1.11) | 0.95 (0.76-1.19) | 1.20 (1.00-1.43) |
|  | Mantle Cell | 1,648,416 | 71 | 0.88 (0.62-1.26) | 1.20 (0.92-1.55) | 1.31 (1.03-1.66) | 1.01 (0.75-1.34) | 0.76 (0.52-1.12) | 0.80 (0.59-1.08) | 1.03 (0.80-1.33) |
|  | Other B-cell | 1,648,416 | 201 | 1.01 (0.82-1.24) | 1.01 (0.85-1.20) | 1.00 (0.85-1.19) | 1.07 (0.91-1.26) | 0.79 (0.63-1.00) | 0.97 (0.82-1.14) | 1.04 (0.90-1.20) |
|  | T-cell Lymphomas | 1,648,416 | 98 | 0.87 (0.64-1.17) | 0.91 (0.70-1.19) | 0.97 (0.76-1.24) | 1.07 (0.84-1.35) | 1.00 (0.72-1.39) | 0.93 (0.73-1.18) | 1.17 (0.96-1.41) |
|  | Other NHL | 1,648,416 | 109 | 0.99 (0.76-1.30) | 0.92 (0.72-1.17) | 0.90 (0.71-1.14) | 0.87 (0.69-1.10) | 1.11 (0.82-1.51) | 1.21 (1.01-1.45) | 0.85 (0.69-1.05) |
|  | Myeloid Leukemias | 1,648,416 | 329 | 0.99 (0.85-1.17) | 0.97 (0.84-1.11) | 0.97 (0.85-1.10) | 0.98 (0.86-1.12) | 1.07 (0.89-1.28) | 1.01 (0.90-1.15) | 0.97 (0.86-1.09) |
|  | AML | 1,648,416 | 223 | 0.98 (0.81-1.19) | 0.94 (0.79-1.12) | 0.94 (0.79-1.10) | 1.00 (0.85-1.17) | 1.01 (0.81-1.26) | 1.06 (0.92-1.23) | 0.98 (0.85-1.13) |
|  | CML | 1,648,416 | 61 | 0.93 (0.64-1.36) | 1.07 (0.79-1.45) | 1.12 (0.85-1.49) | 0.81 (0.58-1.14) | 1.28 (0.83-1.97) | 0.89 (0.65-1.20) | 0.88 (0.66-1.18) |
| **Fully Adjusted ^2^  No Ecologic Data** | Hodgkin Lymphoma | 1,648,416 | 54 | 1.33 (0.92-1.91) | 1.12 (0.83-1.50) | 0.99 (0.72-1.35) | 1.30 (1.00-1.69) | 1.41 (0.90-2.20) | 1.21 (0.94-1.55) | 1.18 (0.94-1.48) |
|  | NHL | 1,648,416 | 2,276 | 0.94 (0.88-1.00) | 0.96 (0.91-1.01) | 0.98 (0.93-1.03) | 0.99 (0.94-1.04) | 0.94 (0.88-1.01) | 0.94 (0.90-0.99) | 1.03 (0.98-1.07) |
|  | B-Cell Lymphomas | 1,648,416 | 2,069 | 0.94 (0.88-1.00) | 0.96 (0.91-1.02) | 0.99 (0.93-1.04) | 1.00 (0.94-1.05) | 0.93 (0.87-1.00) | 0.93 (0.88-0.98) | 1.03 (0.98-1.08) |
|  | DLBCL | 1,648,416 | 473 | 0.94 (0.82-1.08) | 0.98 (0.87-1.10) | 1.01 (0.90-1.12) | 1.04 (0.93-1.16) | 1.04 (0.89-1.21) | 0.94 (0.84-1.05) | 1.08 (0.99-1.19) |
|  | CLL/SLL | 1,648,416 | 507 | 0.89 (0.78-1.01) | 0.89 (0.79-1.01) | 0.93 (0.84-1.04) | 0.89 (0.80-1.00) | 0.94 (0.82-1.09) | 0.90 (0.81-1.00) | 0.95 (0.87-1.05) |
|  | Follicular | 1,648,416 | 269 | 1.01 (0.84-1.20) | 0.98 (0.84-1.15) | 0.98 (0.84-1.13) | 0.98 (0.84-1.14) | 0.99 (0.81-1.20) | 0.94 (0.82-1.08) | 1.05 (0.93-1.19) |
|  | Multiple Myeloma | 1,648,416 | 434 | 0.92 (0.80-1.06) | 0.89 (0.78-1.01) | 0.91 (0.80-1.02) | 1.00 (0.89-1.12) | 0.91 (0.77-1.06) | 0.94 (0.84-1.06) | 1.01 (0.91-1.12) |
|  | Marginal Zone | 1,648,416 | 114 | 0.97 (0.73-1.29) | 1.12 (0.90-1.39) | 1.16 (0.94-1.43) | 1.20 (0.97-1.48) | 0.82 (0.60-1.11) | 0.96 (0.76-1.20) | 1.20 (1.00-1.45) |
|  | Mantle Cell | 1,648,416 | 71 | 0.91 (0.63-1.30) | 1.25 (0.96-1.63) | 1.36 (1.07-1.74) | 1.04 (0.77-1.39) | 0.76 (0.52-1.11) | 0.78 (0.58-1.07) | 1.06 (0.82-1.38) |
|  | Other B-cell | 1,648,416 | 201 | 1.01 (0.82-1.24) | 1.02 (0.85-1.21) | 1.02 (0.86-1.20) | 1.06 (0.90-1.25) | 0.79 (0.63-0.99) | 0.97 (0.82-1.14) | 1.03 (0.89-1.19) |
|  | T-cell Lymphomas | 1,648,416 | 98 | 0.86 (0.64-1.17) | 0.92 (0.70-1.21) | 0.98 (0.77-1.26) | 1.06 (0.83-1.35) | 0.99 (0.71-1.38) | 0.91 (0.71-1.16) | 1.17 (0.96-1.43) |
|  | Other NHL | 1,648,416 | 109 | 0.99 (0.76-1.30) | 0.93 (0.73-1.18) | 0.91 (0.72-1.15) | 0.87 (0.69-1.10) | 1.11 (0.82-1.51) | 1.20 (1.00-1.45) | 0.85 (0.69-1.05) |
|  | Myeloid Leukemias | 1,648,416 | 329 | 1.00 (0.85-1.17) | 0.99 (0.86-1.14) | 0.99 (0.87-1.13) | 0.99 (0.86-1.13) | 1.08 (0.90-1.30) | 1.01 (0.89-1.14) | 0.98 (0.87-1.10) |
|  | AML | 1,648,416 | 223 | 0.98 (0.80-1.19) | 0.95 (0.80-1.13) | 0.95 (0.81-1.13) | 1.00 (0.85-1.18) | 1.01 (0.81-1.27) | 1.05 (0.91-1.23) | 0.99 (0.85-1.14) |
|  | CML | 1,648,416 | 61 | 0.95 (0.65-1.38) | 1.10 (0.82-1.49) | 1.16 (0.87-1.54) | 0.81 (0.58-1.15) | 1.33 (0.86-2.04) | 0.88 (0.65-1.20) | 0.88 (0.65-1.19) |
| 1 Stratified on Age in 1992, adjusted for sex. | | | | | | | | | | |
| 2 Stratified on Age in 1992, adjusted for sex, race, education, marital status, BMI, time varying smoking (years smoked, cigarettes/day, years since quit), started smoking <18, years passive smoking, ACS diet score, alcohol, occupational dirtiness, industrial exposures. | | | | | | | | | | |

Supplemental Table 3. Air pollution models using fixed^1^ air pollution data in association with hematologic cancers (1992-2017).

|  |  |  | **Hazard Ratios (95% Confidence Intervals)^2^** | | | | | | |  |
| --- | --- | --- | --- | --- | --- | --- | --- | --- | --- | --- |
|  |  |  | **PM_2.5_** | **PM_10_** | **PM_10-2.5_** | **NO_2_** | **O_3_** | **SO_2_** | **CO** |  |
| **Cancer Site** | **Person-years** | **Cases** | **per 3.5 μg/m^3^** | **per 6.3 μg/m^3^** | **per 4.8 μg/m^3^** | **per 6.4 ppb** | **per 8.3 ppb** | **per 1.6 ppb** | **per 0.10 ppm** |  |
| Hodgkin Lymphoma | 1,648,416 | 54 | 1.46 (0.97-2.20) | 1.16 (0.84-1.60) | 1.02 (0.73-1.41) | 1.41 (1.05-1.89) | 1.43 (0.88-2.32) | 1.40 (1.07-1.84) | 1.16 (0.95-1.41) |  |
| Non-Hodgkin Lymphomas | 1,648,416 | 2,276 | 0.93 (0.87-0.99) | 0.96 (0.91-1.02) | 0.99 (0.94-1.04) | 0.99 (0.94-1.05) | 0.96 (0.89-1.02) | 0.94 (0.89-0.99) | 1.02 (0.99-1.06) |  |
| B-Cell Lymphomas | 1,648,416 | 2,069 | 0.93 (0.87-1.00) | 0.97 (0.91-1.03) | 0.99 (0.94-1.05) | 0.99 (0.94-1.05) | 0.96 (0.89-1.03) | 0.93 (0.88-0.98) | 1.02 (0.98-1.06) |  |
| DLBCL | 1,648,416 | 473 | 0.93 (0.81-1.07) | 0.98 (0.87-1.10) | 1.01 (0.90-1.13) | 1.03 (0.92-1.16) | 1.08 (0.92-1.26) | 0.95 (0.84-1.07) | 1.06 (0.98-1.14) |  |
| CLL/SLL | 1,648,416 | 507 | 0.85 (0.74-0.97) | 0.89 (0.78-1.00) | 0.94 (0.84-1.05) | 0.88 (0.78-0.99) | 0.96 (0.83-1.11) | 0.88 (0.78-0.99) | 0.95 (0.88-1.03) |  |
| Follicular | 1,648,416 | 269 | 0.93 (0.77-1.13) | 0.98 (0.83-1.15) | 1.00 (0.86-1.17) | 0.94 (0.80-1.10) | 0.98 (0.81-1.20) | 0.92 (0.78-1.07) | 1.00 (0.90-1.11) |  |
| Multiple Myeloma | 1,648,416 | 434 | 0.96 (0.82-1.11) | 0.93 (0.81-1.05) | 0.93 (0.83-1.06) | 1.01 (0.90-1.15) | 0.92 (0.79-1.07) | 0.95 (0.84-1.07) | 1.02 (0.94-1.10) |  |
| Marginal Zone | 1,648,416 | 114 | 0.97 (0.72-1.29) | 1.10 (0.87-1.39) | 1.14 (0.92-1.41) | 1.21 (0.97-1.51) | 0.88 (0.66-1.19) | 1.01 (0.80-1.27) | 1.15 (1.00-1.32) |  |
| Mantle Cell | 1,648,416 | 71 | 0.98 (0.67-1.42) | 1.29 (0.98-1.69) | 1.36 (1.05-1.76) | 1.07 (0.79-1.46) | 0.85 (0.58-1.24) | 0.74 (0.53-1.04) | 1.08 (0.89-1.30) |  |
| Other B-cell | 1,648,416 | 201 | 1.06 (0.85-1.32) | 1.05 (0.87-1.25) | 1.03 (0.86-1.22) | 1.11 (0.93-1.32) | 0.83 (0.67-1.04) | 1.01 (0.84-1.20) | 1.05 (0.93-1.17) |  |
| T-cell Lymphomas | 1,648,416 | 98 | 0.79 (0.57-1.09) | 0.87 (0.66-1.16) | 0.96 (0.74-1.24) | 1.07 (0.83-1.37) | 1.01 (0.72-1.40) | 0.86 (0.66-1.13) | 1.14 (0.97-1.32) |  |
| Other NHL | 1,648,416 | 109 | 0.98 (0.73-1.32) | 0.91 (0.70-1.18) | 0.90 (0.70-1.15) | 0.86 (0.67-1.11) | 0.95 (0.70-1.29) | 1.16 (0.93-1.44) | 0.91 (0.76-1.08) |  |
| Myeloid Leukemias | 1,648,416 | 329 | 0.99 (0.84-1.18) | 1.00 (0.87-1.16) | 1.01 (0.88-1.15) | 1.00 (0.87-1.15) | 1.01 (0.85-1.21) | 0.98 (0.86-1.13) | 1.01 (0.92-1.10) |  |
| AML | 1,648,416 | 223 | 0.99 (0.80-1.22) | 1.00 (0.84-1.19) | 1.00 (0.85-1.18) | 1.02 (0.86-1.21) | 0.91 (0.74-1.13) | 1.01 (0.86-1.19) | 1.03 (0.92-1.15) |  |
| CML | 1,648,416 | 61 | 0.87 (0.58-1.30) | 1.02 (0.73-1.43) | 1.09 (0.80-1.48) | 0.86 (0.60-1.22) | 1.37 (0.88-2.13) | 0.89 (0.64-1.24) | 0.95 (0.75-1.19) |  |
| ^1^ Fixed air pollution data assigns a single average value from CACES for the years 1992-2015 to the baseline census block group for all subjects. | | | | | | | | | |  |
| ^2^Stratified on Age in 1992, adjusted for sex, race, education, marital status, BMI, years smoked, cigarettes/day, years since quit, started smoking <18, years passive smoking, ACS diet score, alcohol, occupational dirtiness, and industrial exposures. Units presented are based on the 5th-mean percentiles when using fixed data. | | | | | | | | | |  |
|  |  |  |  |  |  |  |  |  |  |  |

Supplemental Table 4. Association of particulate matter air pollutants with hematologic subtypes by gender in the CPS-II Nutrition Cohort from 1992-2017.

|  |  |  | |  |  | **Hazard Ratios (95% Confidence Intervals)^1^** | | | | | |
| --- | --- | --- | --- | --- | --- | --- | --- | --- | --- | --- | --- |
|  |  |  | |  |  | **PM_2.5_** | | **PM_10_** | | **PM_10-2.5_** | |
|  | **Person Years** | | **Cases** | | | **per 4.1 mg/m^3^** | | **per 6.7 mg/m^3^** | | **per 5.0 mg/m^3^** | |
| **Cancer Site** | **Men** | **Women** | | **Men** | **Women** | **Men** | **Women** | **Men** | **Women** | **Men** | **Women** |
| Hodgkin Lymphoma | 706,855 | 941,561 | | 23 | 31 | 0.76 (0.39-1.46) | 1.73 (1.06-2.82) | 0.88 (0.46-1.71) | 1.20 (0.81-1.79) | 1.05 (0.58-1.89) | 0.93 (0.60-1.45) |
| Non-Hodgkin Lymphomas | 706,855 | 941,561 | | 1,236 | 1,040 | 0.93 (0.86-1.02) | 0.95 (0.86-1.05) | 0.93 (0.86-1.01) | 0.97 (0.89-1.05) | 0.95 (0.88-1.03) | 0.99 (0.91-1.08) |
| B-Cell Lymphomas | 706,855 | 941,561 | | 1,115 | 954 | 0.94 (0.85-1.02) | 0.95 (0.86-1.04) | 0.94 (0.86-1.02) | 0.96 (0.88-1.05) | 0.96 (0.88-1.04) | 0.98 (0.90-1.07) |
| DLBCL | 706,855 | 941,561 | | 247 | 226 | 0.96 (0.79-1.16) | 0.93 (0.76-1.14) | 0.96 (0.81-1.14) | 0.97 (0.81-1.17) | 0.97 (0.82-1.15) | 1.01 (0.84-1.20) |
| CLL/SLL | 706,855 | 941,561 | | 288 | 219 | 0.92 (0.77-1.10) | 0.83 (0.67-1.02) | 0.88 (0.74-1.03) | 0.84 (0.69-1.03) | 0.89 (0.76-1.04) | 0.91 (0.76-1.10) |
| Follicular | 706,855 | 941,561 | | 125 | 144 | 1.06 (0.81-1.39) | 1.03 (0.81-1.32) | 0.85 (0.65-1.11) | 1.08 (0.87-1.34) | 0.77 (0.59-1.01) | 1.09 (0.88-1.34) |
| Multiple Myeloma | 706,855 | 941,561 | | 239 | 195 | 0.89 (0.73-1.08) | 0.95 (0.76-1.20) | 0.91 (0.76-1.09) | 0.80 (0.64-1.01) | 0.96 (0.81-1.14) | 0.80 (0.64-0.98) |
| Marginal Zone | 706,855 | 941,561 | | 53 | 61 | 1.05 (0.69-1.61) | 0.92 (0.62-1.37) | 1.16 (0.80-1.67) | 1.14 (0.81-1.61) | 1.16 (0.82-1.66) | 1.24 (0.89-1.71) |
| Mantle Cell | 706,855 | 941,561 | | 47 | 24 | 0.76 (0.47-1.23) | 1.21 (0.67-2.18) | 1.26 (0.83-1.91) | 1.30 (0.85-2.00) | 1.53 (1.05-2.22) | 1.32 (0.84-2.06) |
| Other B-cell | 706,855 | 941,561 | | 116 | 85 | 0.93 (0.70-1.23) | 1.09 (0.78-1.52) | 1.00 (0.78-1.30) | 1.07 (0.80-1.43) | 1.06 (0.83-1.35) | 1.04 (0.78-1.38) |
| T-cell Lymphomas | 706,855 | 941,561 | | 57 | 41 | 0.71 (0.46-1.09) | 0.99 (0.61-1.60) | 0.78 (0.51-1.20) | 1.11 (0.73-1.67) | 0.94 (0.65-1.37) | 1.14 (0.76-1.71) |
| Other NHL | 706,855 | 941,561 | | 64 | 45 | 1.08 (0.75-1.56) | 1.02 (0.65-1.59) | 0.95 (0.67-1.35) | 1.04 (0.69-1.57) | 0.88 (0.61-1.25) | 1.04 (0.69-1.56) |
| Myeloid Leukemias | 706,855 | 941,561 | | 195 | 134 | 1.04 (0.83-1.30) | 0.96 (0.74-1.24) | 1.06 (0.87-1.28) | 0.91 (0.71-1.16) | 1.05 (0.87-1.27) | 0.91 (0.72-1.15) |
| AML | 706,855 | 941,561 | | 132 | 91 | 0.98 (0.74-1.28) | 0.99 (0.72-1.36) | 0.96 (0.76-1.22) | 0.92 (0.68-1.24) | 0.97 (0.77-1.22) | 0.90 (0.67-1.21) |
| CML | 706,855 | 941,561 | | 31 | 30 | 1.37 (0.76-2.48) | 0.72 (0.42-1.23) | 1.50 (0.90-2.52) | 0.92 (0.58-1.46) | 1.34 (0.81-2.21) | 1.08 (0.70-1.68) |
| ^1^Stratified on Age in 1992, adjusted for sex, race, education, marital status, BMI, time varying smoking (years smoked, cigarettes/day, years since quit), started smoking <18, years passive smoking, ACS diet score, alcohol, occupational dirtiness, industrial exposures, and census tract data (median household income, % college educated, % African American, % other non-White race, unemployment rate, and poverty rate) | | | | | | | | | | | |

Supplemental Table 5. Association of gaseous air pollutants with hematologic subtypes by gender in the CPS-II Nutrition Cohort from 1992-2017

|  |  |  |  |  | **Hazard Ratios (95% Confidence Intervals)^1^** | | | | | | | |
| --- | --- | --- | --- | --- | --- | --- | --- | --- | --- | --- | --- | --- |
|  |  |  |  |  | **NO_2_** | | **O_3_** | | **SO_2_** | | **CO** | |
|  | **Person Years** | | **Cases** | | **per 7.2 ppb** | | **per 9.9 ppb** | | **per 2.3 ppb** | | **per 0.21 ppm** | |
| **Cancer Site** | **Men** | **Women** | **Men** | **Women** | **Men** | **Women** | **Men** | **Women** | **Men** | **Women** | **Men** | **Women** |
| Hodgkin Lymphoma | 706,855 | 941,561 | 23 | 31 | 1.27 (0.72-2.24) | 1.45 (0.97-2.16) | 0.97 (0.49-1.92) | 1.74 (0.93-3.28) | 1.15 (0.73-1.79) | 1.28 (0.92-1.78) | 1.42 (0.89-2.26) | 1.17 (0.82-1.68) |
| NHL | 706,855 | 941,561 | 1,236 | 1,040 | 0.99 (0.91-1.07) | 1.00 (0.91-1.09) | 0.93 (0.84-1.02) | 0.97 (0.88-1.08) | 0.95 (0.89-1.02) | 0.94 (0.87-1.01) | 1.00 (0.93-1.07) | 1.07 (0.99-1.15) |
| B-Cell Lymphomas | 706,855 | 941,561 | 1,115 | 954 | 0.99 (0.91-1.08) | 0.99 (0.90-1.08) | 0.92 (0.83-1.01) | 0.96 (0.86-1.07) | 0.94 (0.87-1.01) | 0.93 (0.86-1.01) | 1.00 (0.93-1.08) | 1.06 (0.98-1.15) |
| DLBCL | 706,855 | 941,561 | 247 | 226 | 1.05 (0.88-1.24) | 1.00 (0.83-1.21) | 1.10 (0.88-1.37) | 1.01 (0.81-1.27) | 0.95 (0.81-1.10) | 0.98 (0.83-1.15) | 1.07 (0.92-1.24) | 1.07 (0.91-1.26) |
| CLL/SLL | 706,855 | 941,561 | 288 | 219 | 0.87 (0.73-1.03) | 0.80 (0.66-0.98) | 0.86 (0.71-1.05) | 1.05 (0.84-1.31) | 0.91 (0.78-1.04) | 0.89 (0.76-1.05) | 0.91 (0.79-1.06) | 0.91 (0.77-1.08) |
| Follicular | 706,855 | 941,561 | 125 | 144 | 0.94 (0.72-1.22) | 1.10 (0.88-1.37) | 1.07 (0.79-1.44) | 0.99 (0.75-1.31) | 1.10 (0.90-1.33) | 0.83 (0.67-1.03) | 0.95 (0.75-1.19) | 1.23 (1.02-1.49) |
| Multiple Myeloma | 706,855 | 941,561 | 239 | 195 | 1.07 (0.90-1.27) | 0.94 (0.76-1.17) | 0.88 (0.71-1.10) | 0.93 (0.74-1.19) | 0.88 (0.75-1.04) | 1.01 (0.85-1.20) | 1.04 (0.89-1.21) | 1.00 (0.82-1.22) |
| Marginal Zone | 706,855 | 941,561 | 53 | 61 | 1.39 (0.97-1.99) | 1.20 (0.85-1.70) | 0.93 (0.59-1.46) | 0.76 (0.49-1.15) | 0.94 (0.67-1.32) | 0.97 (0.71-1.34) | 1.32 (0.96-1.82) | 1.27 (0.93-1.73) |
| Mantle Cell | 706,855 | 941,561 | 47 | 24 | 1.17 (0.76-1.81) | 0.95 (0.57-1.61) | 0.69 (0.43-1.12) | 0.92 (0.45-1.88) | 0.87 (0.60-1.27) | 0.61 (0.33-1.13) | 1.30 (0.88-1.92) | 0.88 (0.54-1.44) |
| Other B-cell | 706,855 | 941,561 | 116 | 85 | 0.87 (0.66-1.15) | 1.27 (0.96-1.68) | 0.75 (0.56-1.02) | 0.80 (0.57-1.14) | 0.98 (0.79-1.22) | 0.95 (0.73-1.23) | 0.85 (0.66-1.09) | 1.22 (0.95-1.56) |
| T-cell Lymphomas | 706,855 | 941,561 | 57 | 41 | 0.88 (0.59-1.33) | 1.31 (0.87-1.97) | 0.83 (0.54-1.28) | 1.15 (0.68-1.94) | 0.84 (0.60-1.19) | 0.89 (0.60-1.31) | 1.13 (0.81-1.58) | 1.41 (0.99-2.00) |
| Other NHL | 706,855 | 941,561 | 64 | 45 | 1.03 (0.72-1.47) | 0.87 (0.55-1.36) | 1.22 (0.81-1.82) | 1.10 (0.67-1.80) | 1.24 (0.98-1.56) | 1.12 (0.83-1.53) | 0.94 (0.69-1.28) | 0.86 (0.57-1.28) |
| Myeloid Leukemias | 706,855 | 941,561 | 195 | 134 | 1.05 (0.86-1.29) | 0.86 (0.66-1.11) | 1.11 (0.87-1.42) | 1.05 (0.79-1.41) | 1.05 (0.89-1.24) | 0.96 (0.79-1.17) | 1.05 (0.88-1.26) | 0.83 (0.66-1.05) |
| AML | 706,855 | 941,561 | 132 | 91 | 1.03 (0.81-1.31) | 0.88 (0.64-1.20) | 1.01 (0.75-1.36) | 1.03 (0.72-1.46) | 1.10 (0.90-1.34) | 1.03 (0.81-1.31) | 1.06 (0.86-1.32) | 0.78 (0.58-1.05) |
| CML | 706,855 | 941,561 | 31 | 30 | 0.90 (0.49-1.63) | 0.75 (0.44-1.27) | 2.05 (1.05-4.02) | 0.93 (0.50-1.71) | 1.08 (0.71-1.65) | 0.67 (0.40-1.12) | 0.65 (0.34-1.25) | 1.00 (0.66-1.50) |
| ^1^Stratified on Age in 1992, adjusted for sex, race, education, marital status, BMI, time varying smoking (years smoked, cigarettes/day, years since quit), started smoking <18, years passive smoking, ACS diet score, alcohol, occupational dirtiness, industrial exposures, and census tract data (median household income, % college educated, % African American, % other non-White race, unemployment rate, and poverty rate) | | | | | | | | | | | | |

Supplemental Table 6. Association of PM2.5 with hematologic subtypes by US Region in the CPS-II Nutrition Cohort from 1992-2017.

|  | **Northeast** | | **South** | | **Midwest** | | **West** | |
| --- | --- | --- | --- | --- | --- | --- | --- | --- |
| **Cancer Site** | **Cases** | **Hazard Ratio (95% CI)^1^** | **Cases** | **Hazard Ratio (95% CI)^1^** | **Cases** | **Hazard Ratio (95% CI)^1^** | **Cases** | **Hazard Ratio (95% CI)^1^** |
| Hodgkin Lymphoma | 25 | 1.39 (0.74-2.61) | 7 | 0.61 (0.21-1.78) | 13 | 1.44 (0.61-3.42) | 9 | 1.26 (0.64-2.48) |
| Non-Hodgkin Lymphomas | 636 | 0.91 (0.79-1.03) | 417 | 0.98 (0.85-1.15) | 712 | 0.84 (0.74-0.96) | 511 | 1.02 (0.93-1.13) |
| B-Cell Lymphomas | 573 | 0.90 (0.78-1.03) | 367 | 1.00 (0.85-1.17) | 660 | 0.81 (0.71-0.93) | 469 | 1.05 (0.95-1.17) |
| DLBCL | 143 | 0.76 (0.57-1.02) | 79 | 1.17 (0.82-1.66) | 138 | 0.79 (0.59-1.05) | 113 | 1.06 (0.86-1.31) |
| CLL/SLL | 132 | 0.88 (0.66-1.18) | 92 | 0.90 (0.65-1.23) | 167 | 0.79 (0.61-1.03) | 116 | 0.98 (0.80-1.21) |
| Follicular | 74 | 1.14 (0.79-1.66) | 47 | 0.96 (0.61-1.50) | 88 | 0.96 (0.67-1.37) | 60 | 1.11 (0.83-1.47) |
| Multiple Myeloma | 120 | 0.95 (0.70-1.29) | 80 | 1.12 (0.79-1.60) | 140 | 0.71 (0.53-0.96) | 94 | 0.99 (0.78-1.25) |
| Marginal Zone | 34 | 1.17 (0.66-2.05) | 15 | 0.73 (0.32-1.66) | 41 | 0.77 (0.44-1.33) | 24 | 1.16 (0.73-1.83) |
| Mantle Cell | 15 | 0.24 (0.08-0.72) | 15 | 0.79 (0.34-1.83) | 23 | 1.14 (0.56-2.33) | 18 | 1.41 (0.85-2.33) |
| Other B-cell | 55 | 1.00 (0.65-1.56) | 39 | 0.91 (0.56-1.49) | 63 | 0.90 (0.59-1.38) | 44 | 1.12 (0.80-1.58) |
| T-cell Lymphomas | 32 | 0.64 (0.35-1.18) | 22 | 0.64 (0.33-1.26) | 21 | 1.23 (0.59-2.56) | 23 | 0.93 (0.56-1.54) |
| Other NHL | 31 | 1.37 (0.80-2.35) | 28 | 1.19 (0.69-2.04) | 31 | 1.29 (0.73-2.28) | 19 | 0.59 (0.33-1.06) |
| Myeloid Leukemias | 91 | 1.15 (0.82-1.61) | 69 | 0.94 (0.65-1.37) | 93 | 1.18 (0.83-1.67) | 76 | 0.87 (0.67-1.15) |
| AML | 62 | 1.20 (0.79-1.82) | 49 | 0.80 (0.52-1.26) | 65 | 1.13 (0.74-1.72) | 47 | 0.86 (0.61-1.21) |
| CML | 16 | 1.08 (0.49-2.37) | 14 | 1.38 (0.60-3.17) | 12 | 1.65 (0.64-4.28) | 19 | 0.63 (0.34-1.16) |

^1^Stratified on Age in 1992, adjusted for sex, race, education, marital status, BMI, time varying smoking (years smoked, cigarettes/day, years since quit), started smoking <18, years passive smoking, ACS diet score, alcohol, occupational dirtiness, industrial exposures, census tract data (median household income, % college educated, % African American, % other non-White race, unemployment rate, and poverty rate), and includes interaction terms for US region.

Supplemental Table 7. Two-pollutant associations with select hematologic subtypes in the CPS-II Nutrition Cohort from 1992-2017

|  |  |  |  | **Hazard Ratios (95% CI)^1^** | | | | |
| --- | --- | --- | --- | --- | --- | --- | --- | --- |
|  |  |  |  | **PM_2.5_** | **PM_10_** | **PM_10-2.5_** | **NO_2_** | **CO** |
| **Cancer Site** | **Pollutants in Model** | **Population** | **Cases** | **per 4.1 mg/m^3^** | **per 6.7 mg/m^3^** | **per 5.0 mg/m^3^** | **per 7.2 ppb** | **per 0.21 ppm** |
| Hodgkin Lymphoma | PM2.5 and NO2 | All | 54 | 1.09 (0.69-1.72) |  |  | ***1.34 (0.91-1.97)*** |  |
|  | PM2.5 and NO2 | Never Smokers | 24 | ***1.72 (0.90-3.32)*** |  |  | 1.22 (0.68-2.22) |  |
| Marginal Zone | PM2.5 and NO2 | All | 114 | 0.77 (0.55-1.10) |  |  | ***1.46 (1.09-1.97)*** |  |
|  | PM2.5 and CO | All | 114 | 0.89 (0.66-1.20) |  |  |  | ***1.33 (1.06-1.69)*** |
|  | NO2 and CO | All | 114 |  |  |  | ***1.09 (0.73-1.63)*** | ***1.22 (0.85-1.76)*** |
| Mantle Cell | PM2.5 and PM10-2.5 | All | 71 | 0.95 (0.72-1.27) |  | ***1.20 (0.94-1.52)*** |  |  |
|  | PM10-2.5 and CO | All | 71 |  |  | ***1.05 (0.79-1.39)*** |  | 1.26 (0.97-1.64) |
| Follicular | PM2.5 and CO | Women | 144 | 0.95 (0.73-1.23) |  |  |  | ***1.25 (1.03-1.52)*** |
|  | PM10 and CO | Women | 144 |  | 0.93 (0.72-1.21) |  |  | ***1.28 (1.02-1.60)*** |
|  | NO2 and CO | Women | 144 |  |  |  | 0.78 (0.56-1.11) | ***1.45 (1.08-1.96)*** |
| T-cell Lymphoma | PM2.5 and CO | All | 98 | 0.77 (0.56-1.08) |  |  |  | ***1.34 (1.04-1.72)*** |
|  | PM10 and CO | All | 98 |  | 0.75 (0.54-1.06) |  |  | ***1.45 (1.09-1.94)*** |
|  | NO2 and CO | All | 98 |  |  |  | 0.75 (0.49-1.13) | ***1.54 (1.08-2.22)*** |

^1^Stratified on Age in 1992, adjusted for sex, race, education, marital status, BMI, time varying smoking (years smoked, cigarettes/day, years since quit), started smoking <18, years passive smoking, ACS diet score, alcohol, occupational dirtiness, industrial exposures, and census tract data (median household income, % college educated, % African American, % other non-White race, unemployment rate, and poverty rate). Italicized and bolded results were statistically significant in single pollutant models.

Supplemental Table 8. PM 2.5 models using alternative data^1^ in association with hematologic cancers from 1992-2017.

|  |  |  | **Hazard Ratios (95% Confidence Intervals)^2^** | | |  |
| --- | --- | --- | --- | --- | --- | --- |
|  |  |  | **PM_2.5_** | **Near Source PM_2.5_** | **Regional PM_2.5_** |  |
| **Cancer Site** | **Person-years** | **Cases** | **per 3.8 mg/m^3^** | **per 1.7 mg/m^3^** | **per 3.5 mg/m^3^** |  |
| Hodgkin Lymphoma | 1,590,996 | 52 | 1.32 (0.93-1.87) | 1.33 (0.80-2.20) | 1.26 (0.89-1.80) |  |
| Non-Hodgkin Lymphomas | 1,590,996 | 2,187 | 0.93 (0.88-0.99) | 0.95 (0.88-1.03) | 0.94 (0.89-1.00) |  |
| B-Cell Lymphomas | 1,590,996 | 1,991 | 0.94 (0.88-1.00) | 0.95 (0.87-1.03) | 0.95 (0.89-1.01) |  |
| DLBCL | 1,590,996 | 455 | 0.93 (0.81-1.06) | 0.86 (0.73-1.02) | 0.96 (0.84-1.09) |  |
| CLL/SLL | 1,590,996 | 492 | 0.83 (0.72-0.94) | 0.85 (0.73-1.00) | 0.86 (0.75-0.97) |  |
| Follicular | 1,590,996 | 256 | 0.96 (0.80-1.14) | 0.95 (0.76-1.19) | 0.97 (0.81-1.15) |  |
| Multiple Myeloma | 1,590,996 | 416 | 0.98 (0.85-1.12) | 1.08 (0.91-1.29) | 0.95 (0.83-1.09) |  |
| Marginal Zone | 1,590,996 | 109 | 0.97 (0.74-1.26) | 1.18 (0.83-1.67) | 0.92 (0.71-1.20) |  |
| Mantle Cell | 1,590,996 | 68 | 1.15 (0.82-1.60) | 1.30 (0.84-2.03) | 1.08 (0.77-1.51) |  |
| Other B-cell | 1,590,996 | 195 | 1.08 (0.89-1.31) | 0.93 (0.72-1.20) | 1.10 (0.91-1.34) |  |
| T-cell Lymphomas | 1,590,996 | 94 | 0.77 (0.57-1.05) | 1.11 (0.77-1.61) | 0.74 (0.54-1.00) |  |
| Other NHL | 1,590,996 | 102 | 1.00 (0.76-1.31) | 0.88 (0.62-1.26) | 1.04 (0.79-1.35) |  |
| Myeloid Leukemias | 1,590,996 | 313 | 0.95 (0.81-1.11) | 0.97 (0.80-1.19) | 0.96 (0.82-1.12) |  |
| AML | 1,590,996 | 211 | 0.94 (0.78-1.14) | 0.97 (0.76-1.24) | 0.95 (0.78-1.15) |  |
| CML | 1,590,996 | 58 | 0.89 (0.61-1.30) | 1.13 (0.70-1.81) | 0.85 (0.59-1.24) |  |
| ^1^Alternative PM2.5 data described in Turner et al. 2017 from 1999-2004 assigned at baseline address. | | | | | |  |
| ^2^Stratified on Age in 1992, adjusted for sex, race, education, marital status, BMI, time varying smoking (years smoked, cigarettes/day, years since quit), started smoking <18, years passive smoking, ACS diet score, alcohol, occupational dirtiness, and industrial exposures. Units are based on 5th-mean percentile for the alternative exposures. | | | | | |  |
|  |  |  |  |  |  |  |
